# First Report of Survival in Refractory Ventricular Fibrillation After Dual-Axis Defibrillation and Esmolol Administration

**DOI:** 10.5811/westjem.2016.8.30351

**Published:** 2016-10-20

**Authors:** Kevin M. Boehm, Daniel C. Keyes, Laura E Mader, J. Michelle Moccia

**Affiliations:** * St. Mary Mercy Hospital, Departments of Graduate Medical Education and Emergency Medicine, Livonia, Michigan; † Michigan State University, College of Osteopathic Medicine, Department of Osteopathic Medical Specialties, East Lansing, Michigan

## Abstract

There is a subset of patients who suffer a witnessed ventricular fibrillation (VF) arrest and despite receiving reasonable care with medications (epinephrine and amiodarone) and multiple defibrillations (3+ attempts at 200 joules of biphasic current) remain in refractory VF (RVF), also known as electrical storm. The mortality for these patients is as high as 97%. We present the case of a patient who, with a novel approach, survived RVF to outpatient follow up.

## INTRODUCTION

Ventricular fibrillation (VF) is a potentially fatal dysrhythmia associated with acute myocardial infarction.^1^ It is well accepted that the longer a patient has to wait for defibrillation, the higher the risk of mortality.^1^ Patients who suffer VF have a decreased risk of mortality with early, definitive care.^2^ However, there is a subset of patients with VF arrest who remain in VF refractory ventricular fibrillation (RVF) despite standard pharmachotherapy (epinephrine and amiodarone) and multiple defibrillations (three or more attempts at 200 joules (J) of biphasic current, also known as *electrical storm*.^3^ The mortality for these patients can be as high as 97%.^3^ We present the case of a patient who received a novel approach to treatment and survived electrical storm to discharge and successful outpatient follow up.

## CASE REPORT

A 67-year-old, 85 kg man with a prior history of left anterior descending artery (LAD) stent placement was brought by emergency medical services (EMS) to the emergency department (ED) of an academic, community-based hospital. He complained of numbness in his left arm that radiated into his chest. He took 325 mg of aspirin 20 minutes prior to EMS arrival, and EMS gave a single 0.4 mg sublingual nitroglycerin while in transport with full relief of pain. Electrocardiogram (ECG) performed by EMS showed normal sinus rhythm. As the patient was undergoing his initial nursing assessment, he reported that he “felt funny;” his upper extremities began to shake, and then he became unresponsive with agonal respirations, followed by apnea. At this point, no pulse was present and the monitor displayed VF. Chest compressions were started and he received biphasic defibrillation at a dose of 200 J. The first attempt at intubation was esophageal, so the endotracheal tube was promptly removed and ventilation resumed via bag-valve mask (BVM) with excellent chest wall rise. The resuscitation continued with administration of epinephrine 1 mg intravenous bolus approximately every three minutes with four total doses given. In addition, he received a total of 450 mg of amiodarone. The patient received a total of five defibrillation shocks, the first four at 200 J and the fifth at 300 J, biphasic.

**Figure 1:**
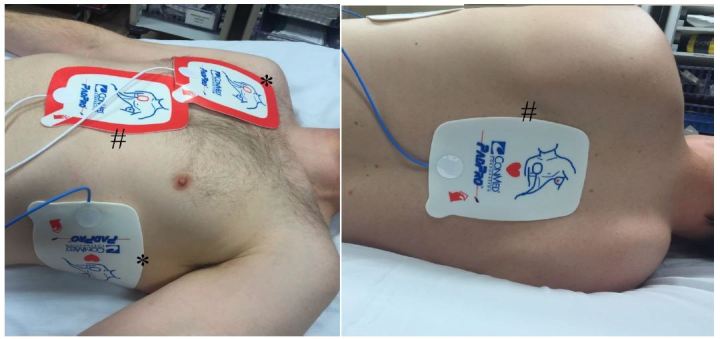
Reproduction of pad placement for dual-axis shock. Pads marked with the asterix (*) show the standard placement of pads, whereas the pads marked with the octothorp (#) signify the anterior-posterior placement for the second set of pads.

After failing to successfully terminate the VF in the first 15 minutes, it was decided to attempt dual axis defibrillation and esmolol administration, so a second defibrillator was brought to the room. A STAT request was made for pharmacy to send esmolol. The paddles of the second defibrillator were placed in an anterior – posterior central position (Figure 1). Coordination of dual discharge using the original pads and the additional second device and pads occurred “on the count of 3,” and 300 J were simultaneously delivered from each device in the 15^th^ minute of resuscitation. There was no change from VF with this intervention, so CPR continued. While cardiopulmonary resuscitation (CPR) was performed, the patient received a bolus of 80 mg of esmolol IV push and an infusion of 0.1 mg/kg/hr was initiated at the 18^th^ minute of the resuscitation attempt. After allowing time for the esmolol to circulate with CPR, there was persistent VF, and a second simultaneous dual defibrillatory shock was delivered after 21 minutes of resuscitation in the same manner as the first. With that attempt, there was return of spontaneous circulation with a room air pulse oximetry of greater than 90%.

**Figure 2:**
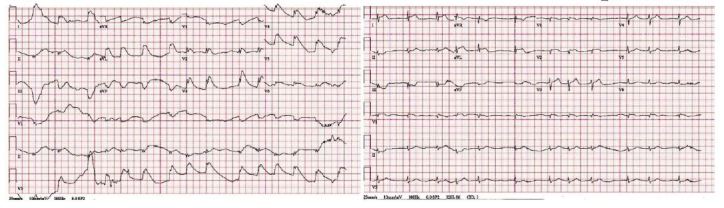
To the left, the electrocardiogram (ECG) immediately following resuscitation. To the right, the ECG approximately 30 minutes after resuscitation.

A second attempt at intubation was initiated at the 23^rd^ minute of the resuscitation attempt, but was aborted when the patient became more alert with the insertion of the laryngoscope. Following resuscitation, the ECG demonstrated atrial fibrillation with 2–5 mm ST elevations in leads I, AVL, and V2–V6 consistent with an ST-segment elevation myocardial infarction (STEMI). Laboratory analysis showed a mild hypokalemia, mild elevation in Troponin I, and mild anemia. A repeat ECG approximately 30 minutes after the resuscitation, and just prior to going to the catheterization laboratory, was still consistent with a STEMI (Figure 2). At this time the patient was awake, speaking in full sentences, and was breathing room air with stable vital signs. He received a heparin bolus and drip and was successfully transferred to the catheterization laboratory where, with minimal sedation, he was found to have a mid-left anterior descending (LAD) lesion. A drug-eluting stent was placed.

The patient had an otherwise uneventful inpatient stay and was discharged on hospital day 4. He was seen again one week later in the outpatient cardiology clinic. He complained of some chest wall soreness, and mild dyspnea on exertion, but otherwise felt well. At follow up with the patient after completion of cardiac rehabilitation, he had no known long-term sequela and was riding his bicycle over eight miles a day. He provided permission for this case report.

## DISCUSSION

The American Heart Association last updated their recommendations for the treatment of VF in 2015.^4^ These guidelines recommend the use of well-performed CPR, initial supplemental oxygen via BVM with consideration of advanced airway management via endotracheal intubation or supraglottic airway device, defibrillation, epinephrine, and amiodarone. The guidelines also make reference to considering the reversible causes, known as the 5 H’s and T’s (hypovolemia, hypoxia, hydrogen ion [acidosis], hyper-/hypokalemia, hypothermia, toxins, tamponade (cardiac), tension pneumothorax, coronary thrombosis and pulmonary thrombosis).^4^ The case presented goes beyond these guidelines, and may be described as a refractory case of VF secondary to electrical storm.^5^–^6^ The current case failed to respond to this standard approach to therapy. As a result, the approach to treatment went beyond these guidelines. Part of the problem with trying to define a treatment for RVF, or electrical storm, is that the formal definition of these conditions are still in debate.^5^–^7^ As early as 2000, “electrical storm” was described as multiple bouts of VF that required not only multiple attempts at defibrillation, but sympathetic blockade in addition to antiarrhythmic pharmacotherapy.^7^ We propose that using the term RVF in the context of resuscitation will allow practitioners to move beyond the standard Advanced Cardiac Life Support (ACLS) guidelines for this almost universally fatal condition^4^ and think about other ways to care for the patient in these circumstances. Although this proposal will exclude even more rare cases of RVF, such as premature ventricular contractions (PVCs) or Brugada syndrome which require completely different treatment strategies, the most common cause of RVF is ischemia.^3^

The use of dual-axis shock is not a new concept in the treatment of RVF. Hoch described five cases of double-axis external shocks as a successful intervention for RVF as early as 1994.^8^ These cases were all performed in the electrophysiology suite, had standard single-axis defibrillator shocks administered over 20 times without success, but were converted back to a normal sinus rhythm after dual-axis defibrillation.^8^ In 2013, Leacock described the first case of successful RVF conversion in the ED after failure of ACLS protocols with two dual-axis defibrillation shocks.^9^ In 2015, Cabañas reported on 10 cases of refractory VF treated with double-axis external defibrillation in the prehospital setting. Three of these patients had return of spontaneous circulation (ROSC), but none survived to discharge with their protocols.^10^ Although the guidelines call for no higher than 200J of biphasic energy and 360J of monophasic energy, multiple studies have shown no ill effects with higher dose shocks, even as high as 720 J (monophasic) delivered using two defibrillators.^9^

It is thought that electrical storm leading to RVF is beta-adrenergic myocardial hyperstimulation that can lower VF threshold and widen ischemic injury. In the setting of cardiac arrest, the patient not only has a swell of endogenous catecholamines, but is also receiving exogenous epinephrine every 3–5 minutes.^6^ Several studies report on survival with positive neurological outcome through the use of standard class III antiarrhythmics with subsequent administration of short-acting beta blockers.^6^–^7^, ^11^–^12^

This case is unique in reporting successful treatment of RVF with the combination of dual-axis shock with beta-blockade. McGovern and McNamee proposed this combination in 2015, wherein a sequence of standard ACLS treatment is followed by simultaneous dual defibrillation from two different axes across the chest, then esmolol, and finally a repeat dual shock.^13^

This index case describes the first successful use of dualaxis defibrillation and esmolol administration with the patient surviving to hospital discharge and outpatient follow up, and we urge more study in its use in an attempt to delineate correlation versus causation. By recognizing RVF early in the resuscitation process, we may be able to deliver a dual-axis shock sooner and also stabilize the myocardium with beta-blockade.
